# Factors associated with perceptions of science communication in Latin America during the COVID-19 pandemic

**DOI:** 10.3389/fpubh.2026.1853486

**Published:** 2026-08-20

**Authors:** Christian R. Mejia, Dafna J. Mendoza, Leticia Rivera-Berrios, Víctor Serna-Alarcón, Leonel Vega, Maria J. Erazo-Muñoz, Camilo Vega-Useche

**Affiliations:** 1 Universidad de Huánuco, Huánuco, Peru; 2 Asociación Médica de Investigación y Servicios en Salud, Lima, Peru; 3 School of Human Medicine, Private University Antenor Orrego, Trujillo, Peru; 4 Hospital de la Amistad Perú Corea Santa Rosa II-2, Piura, Peru; 5 Universidad del Rosario, Bogotá, Colombia; 6 Universidad Pedagógica y Tecnológica de Colombia, Tunja, Colombia; 7 Fundación Santa Fe de Bogotá, Bogotá, Colombia

**Keywords:** COVID-19, cross-sectional study, health information, infodemic, Latin America, science communication

## Abstract

**Background:**

During the COVID-19 pandemic, science communication became central to the public health response amid misinformation, information overload, and rapidly evolving evidence. However, factors associated with perceptions of science communication in Latin America remain insufficiently studied.

**Objective:**

To identify factors associated with a relatively more favorable perception category related to science communication among adults in Latin America during the COVID-19 pandemic.

**Methods:**

We conducted a multicenter cross-sectional analytical study using data from an online survey administered between June and August 2020 in Latin America. Perceptions and experiences related to science communication were assessed using an 11-item Spanish-language questionnaire developed for this study through literature review, expert review, a Delphi-informed process, and pilot testing. Items were scored on a five-point Likert scale, yielding total scores from 11 to 55. The upper tertile of the observed score distribution was defined analytically as the relatively more favorable perception category. Internal consistency was assessed with Cronbach’s alpha. Crude and adjusted prevalence ratios (PRs) with 95% confidence intervals (95% CIs) were estimated using Poisson-family generalized linear models with log link and robust variance.

**Results:**

A total of 9,608 participants from 12 country categories were included. Cronbach’s alpha was 0.833. Overall, 33.3% were classified in the relatively more favorable perception category, reflecting an analytical classification rather than a population prevalence estimate. In adjusted analyses, men had a lower prevalence of this category than women (aPR = 0.88; 95% CI: 0.83–0.94). Primary/secondary and technical education were associated with higher prevalence compared with no formal education. Compared with Peru, Paraguay and Costa Rica showed higher prevalences, whereas Colombia, Bolivia, Ecuador, and Other countries showed lower prevalences.

**Conclusion:**

Perceptions related to science communication were socially patterned by sex, educational level, and country of residence. Given the exploratory instrument and analytical outcome definition, these findings should be interpreted as hypothesis-generating.

## Introduction

Science communication in public health should not be understood as a merely unidirectional process of transmitting data, but rather as a multidimensional process through which scientific knowledge is communicated, interpreted, and socially appropriated by different publics. Contemporary definitions emphasize that science communication involves the use of appropriate skills, media, activities, and dialogue to foster awareness, interest, opinion formation, and understanding of science (1). Its effectiveness depends not only on the technical competence of those who communicate science, but also on the trust they inspire in the public, particularly in contexts marked by high uncertainty and rapidly evolving knowledge (2). This perspective moves beyond the traditional deficit model, which assumes that public responses can be improved simply by transmitting more information (3). This premise became especially relevant during the COVID-19 pandemic, when scientific production, public health decision-making, media coverage, and public debate progressed simultaneously and often at uneven speeds.

Alongside the spread of SARS-CoV-2, an infodemic emerged, understood as an overabundance of information, whether accurate or not, that makes it difficult for people to identify trustworthy content and make appropriate decisions to protect their health (4, 5). The World Health Organization characterized this phenomenon as a major public health challenge and proposed infodemic management as a coordinated response involving monitoring, promoting reliable information, and mitigating the harms of misinformation and disinformation (5). In this context, social media and other digital environments accelerated the circulation of messages about prevention, treatment, the origin of the virus, and public policies, amplifying both evidence-based information and content that was erroneous, misleading, or unverified (6, 7). The pandemic also highlighted the distinction between information overload, misinformation, and intentional disinformation, as well as the role of digital platforms in shaping risk perception and public responses (7). The available evidence has shown that health misinformation during the pandemic was associated with confusion, institutional distrust, lower adherence to public health recommendations, and decisions potentially harmful to individual and collective health (8).

For this reason, the quality of public science communication became a central component of the response to the crisis. A rapid systematic review of public health messaging in infectious disease contexts showed that population responses improve when messages are consistent, clear, transparent about uncertainty, adapted to different population groups, and delivered by sources perceived as credible (9). Risk communication analyses during COVID-19 similarly emphasize that transparent communication about uncertainty is essential to sustain trust when evidence is rapidly evolving (10). However, the pandemic also revealed that trust in science and institutions may weaken when evidence is communicated in contexts of political contestation, publicly modeled uncertainty, or contradictory messaging (11). At the same time, other studies have shown that, even in times of crisis, science may serve as a cognitive and social anchor, provided that the public perceives communicators as legitimate, competent, and acting in good faith (12).

The ways in which individuals seek and evaluate information also play a decisive role in how scientific messages are received. During the pandemic, social media became a frequent source of health information for broad segments of the population (13), while trust in different information sources was associated with the adoption of preventive behaviors (14). Multicountry evidence among younger adults has also shown that social media was a major source of COVID-19 information and that many users felt overwhelmed by pandemic-related digital information, highlighting the importance of digital literacy in interpreting health messages (15). Likewise, international multicenter studies found that susceptibility to COVID-19 misinformation was associated with lower ability to identify false content and with less favorable attitudes toward public health recommendations (16). Although strategies such as psychological inoculation, early correction, and rapid response on social media were proposed to mitigate these effects (17, 18), synthesized evidence suggests that the effectiveness of misinformation countermeasures has been heterogeneous and, in many cases, modest (19). Therefore, it is not enough to study the circulation of false information alone; it is also necessary to understand how individuals appraise, interpret, and perceive the science communication they receive in emergency settings.

These tensions were particularly relevant in Latin America and the Caribbean, a region characterized by profound social inequalities, segmented and fragmented health systems, and heterogeneous institutional capacities to respond to complex public health crises (20, 21). Such conditions not only influenced the magnitude of the pandemic’s impact, but also shaped the ways in which people accessed, interpreted, and evaluated scientific information. Indeed, recent analyses based on the Global Burden of Disease Study 2021 have shown that the burden of COVID-19 in Latin America and the Caribbean was substantial and unequally distributed across countries, reinforcing the need to better understand the social and contextual factors that shape the reception of public health messages in the region (22).

Despite the rapid growth of the COVID-19 literature, much of the available research has focused on misinformation, trust in specific sources, information-seeking behaviors, or preventive practices, rather than on the broader assessment of perceptions of science communication as a social and public health phenomenon (8, 9, 13, 14, 16–19). This gap is especially relevant in Latin America, where information ecosystems, institutional trust, and structural conditions differ markedly across countries (20–22). In this context, this study addressed the following research question: which sociodemographic and contextual factors were associated with a relatively more favorable perception category related to science communication among adults in Latin America during the COVID-19 pandemic? Based on prior evidence on trust, health literacy, information-seeking behavior, and misinformation, we expected perceptions of science communication to vary according to sex, educational level, and country of residence. Therefore, the objective of this study was to identify factors associated with a relatively more favorable perception category related to science communication among adults in Latin America during the COVID-19 pandemic.

## Materials and methods

### Study design

We conducted a multicenter cross-sectional analytical study using data from an online survey administered between June and August 2020, after prospective approval of the parent project, during the first wave of the COVID-19 pandemic in Latin America.

### Population and setting

The target population comprised Spanish-speaking adults residing in Latin American countries during the COVID-19 pandemic. For analytical purposes, participants were classified according to country of residence as Peru, Chile, Paraguay, Mexico, Colombia, Bolivia, Panama, Ecuador, Costa Rica, El Salvador, Honduras, and a grouped category labeled “Other countries” for respondents from countries with smaller sample sizes.

### Sampling strategy and participants

A non-probabilistic snowball sampling strategy was used. In the first stage, the survey was distributed through the authors’ direct contacts and through the network of the Latin American Federation of Scientific Medical Student Societies (FELSOCEM, by its Spanish acronym). Participants were subsequently encouraged to share the questionnaire within their personal networks.

Individuals were eligible if they were aged 18 years or older, resided in a Latin American country, and voluntarily agreed to participate in the study. We excluded respondents who were younger than 18 years, submitted incomplete questionnaires, or showed evidence of inconsistent or patterned responses during data cleaning.

### Variables and instrument

The main outcome was membership in the relatively more favorable perception category derived from an exploratory composite score of perceptions and experiences related to science communication during the COVID-19 pandemic. To explore these perceptions and experiences, we used an 11-item online questionnaire in Spanish developed by the research team specifically for this study. Item generation was informed by the literature available at the time of study design on science communication, trust in scientific information, misinformation, and public responses to COVID-19-related messages. The conceptual basis of the questionnaire drew on prior work addressing trust in science communication, the circulation of misinformation in digital environments, public trust in science under uncertainty, susceptibility to misinformation, and early strategies to counter false information (1, 3, 6, 10, 11, 16–18). Based on this review, a preliminary version of the questionnaire was drafted and subsequently refined through a Delphi-informed process. The instrument then underwent expert review, and the observations received were incorporated into a revised version. A pilot application was conducted before final implementation of the survey. The complete questionnaire, scoring rules, response options, analytical categorization, and conceptual mapping of items are provided in Supplementary Material 1.

All 11 items were scored according to the original coding scheme defined by the research team. Each item used a five-point Likert-type response scale with the following response options: strongly disagree, disagree, neither agree nor disagree, agree, and strongly agree. Responses were coded from 1 to 5, and the total score was calculated as the sum of the 11 items, yielding a possible range from 11 to 55 points. No items were reverse-coded in the main scoring scheme. Although some items referred to exposure to false or dubious information, pessimistic media coverage, or perceived harm from media information, the original scoring scheme was retained because all items showed positive item-test correlations and positive loadings on the first factor in the exploratory factor analysis. Therefore, the total score was interpreted as an exploratory composite measure rather than as a fully validated unidimensional scale.

For the main regression analyses, the total score was categorized for analytical purposes using the tertile rule. Participants whose total score fell within the upper tertile of the observed distribution were classified as belonging to the relatively more favorable perception category, whereas those in the middle and lower tertiles were grouped into the less favorable perception category. Because no validated cut-off point was available for this questionnaire, the upper tertile was used as an analytical threshold to identify participants with relatively higher scores. This threshold should not be interpreted as a clinically, psychometrically, or population-validated definition of favorable perception.

The main independent variables were sex, age, educational level, country of residence, and self-reported history of COVID-19. Educational level was grouped into no formal education, primary/secondary education, technical education, and higher education. Self-reported history of COVID-19 was analyzed as a binary variable. Countries with smaller sample sizes were grouped into an “Other countries” category for regression analyses.

### Procedures

The questionnaire was administered online between June and August 2020. Before answering the survey, participants were informed about the study objectives and the voluntary nature of participation. Completion of the questionnaire was considered evidence of informed consent. No personally identifiable information was collected. After data collection, responses were exported to Microsoft Excel for initial cleaning and then analyzed in StataNow version 19.5.

### Statistical analysis

Categorical variables were summarized as absolute frequencies and percentages. Because age did not follow a normal distribution, it was described using the median and interquartile range (IQR). The distribution of the total composite score was summarized using mean, standard deviation, median, IQR, and selected percentiles.

Internal consistency of the 11-item questionnaire was assessed using Cronbach’s alpha. As an item-level diagnostic, Cronbach’s alpha if item deleted was examined to explore the contribution of each item to the overall internal consistency of the questionnaire. Given that the questionnaire was specifically developed for this study, its psychometric assessment was considered exploratory. To provide additional evidence on dimensionality, an exploratory factor analysis was conducted using principal-component factors. Factor loadings, eigenvalues, and the proportion of variance explained were examined. This analysis was intended only as an exploratory assessment of the internal structure of the questionnaire. No test–retest reliability or cross-country measurement invariance analyses were performed.

Only complete questionnaires were included in the analytical dataset. Participants with missing data in any of the 11 questionnaire items, country of residence, sex, age, educational level, or self-reported COVID-19 history were excluded. Participants younger than 18 years were also excluded. No imputation procedures were performed.

In the bivariate analysis, the distribution of the relatively more favorable and less favorable perception categories was compared across categorical covariates using the chi-square test, whereas age was compared using the Wilcoxon rank-sum test.

For the multivariable analysis, crude and adjusted prevalence ratios (PRs) with 95% confidence intervals (95% CIs) were estimated using generalized linear models from the Poisson family with log link and robust variance. Country of residence was included as a fixed covariate in the regression models, with Peru as the reference category. Robust standard errors were used. Variables were included in the adjusted model *a priori* based on conceptual relevance and previous literature, rather than through data-driven selection procedures. Age was modeled per 10-year increase to improve interpretability.

Sensitivity analyses were conducted to assess the robustness of the findings. These included modeling the total score as a continuous outcome using linear regression with robust standard errors, repeating the Poisson model after excluding Peru, and using alternative binary definitions of higher scores based on the median and the 75th percentile. The full exploratory factor analysis results and sensitivity analyses are provided in Supplementary Material 2. A *p*-value <0.05 was considered statistically significant.

### Ethical considerations

This study was conducted as a secondary analysis of an anonymized database derived from a parent COVID-19 research project. The parent project was prospectively reviewed and approved by the Bioethics Committee of Universidad Privada Antenor Orrego (UPAO) under Resolution No. 0239-2020-UPAO, dated June 5, 2020. The present secondary analysis was subsequently reviewed and approved by the same Bioethics Committee under Resolution No. 0020-2022-UPAO, dated February 11, 2022. The study was conducted in accordance with the ethical principles of the Declaration of Helsinki. Before answering the survey, participants were informed about the study objectives and the voluntary nature of participation. Completion of the questionnaire was considered evidence of informed consent. No personally identifiable information was collected.

## Results

A total of 9,608 participants were included in the analysis and completed the 11-item questionnaire assessing perceptions and experiences related to science communication during the pandemic. The questionnaire showed acceptable internal consistency in this sample, with an overall Cronbach’s alpha of 0.833. Item-level diagnostics, expressed as Cronbach’s alpha if item deleted, are presented in Table 1. Given the exploratory nature of the instrument, these findings should be interpreted as preliminary measurement evidence. In the exploratory factor analysis, the first factor had an eigenvalue of 4.31 and explained 39.2% of the variance. Three factors had eigenvalues greater than 1 and together explained 62.1% of the variance, supporting the interpretation of the questionnaire as an exploratory composite measure rather than as a fully validated unidimensional scale.

**Table 1:** Item content and internal consistency diagnostics of the 11-item questionnaire assessing perceptions related to science communication during the COVID-19 pandemic.

Item	Questionnaire item	Cronbach’s alpha if item deleted
1	Do you think that the information being disseminated about supposed remedies or cures for the coronavirus is based on verified scientific sources?	0.839
2	Do you think that the information being disseminated about how the coronavirus originated is based on verified scientific sources?	0.827
3	Have you come across false or dubious scientific information about the coronavirus?	0.818
4	Do you believe that the news media provide adequate scientific information about COVID-19?	0.824
5	Do you think the language used by online media to inform the public is understandable?	0.815
6	Based on what the media shows you daily, do you perceive that most news is bad or pessimistic?	0.822
7	Would you like to have a source that confirms or refutes the news you hear or see in the media?	0.810
8	Do you believe the doctors interviewed helped you understand/clarify the scientific news about COVID-19?	0.803
9	Do you believe that the other scientists interviewed helped you understand/clarify the scientific news about COVID-19?	0.802
10	Has the scientific information provided by the government been useful in combating the coronavirus?	0.815
11	Did the information provided by the media affect you or cause you any problems or harm at any point during the pandemic?	0.834

Cronbach’s alpha for the overall 11-item questionnaire was 0.833. Item-level values correspond to Cronbach’s alpha if item deleted and should not be interpreted as reliability coefficients for individual items. All items were scored according to the original coding scheme defined by the research team. Response options were: strongly disagree, disagree, neither agree nor disagree, agree, and strongly agree. Given the exploratory nature of the instrument, these internal consistency findings should be interpreted as preliminary measurement evidence.

Participants ranged in age from 18 to 89 years, with a median age of 22 years (interquartile range [IQR]: 20–29). Women accounted for 59.5% of the sample, 78.0% had higher education, 86.0% had technical or higher education, and 97.0% reported no history of COVID-19. Peru contributed 49.3% of the total sample. According to the predefined tertile-based classification, 33.3% of participants were categorized as belonging to the relatively more favorable perception category related to science communication (Table 2). Because this category was defined using the upper tertile of the observed score distribution, this proportion should be interpreted as an analytical classification rather than as a population prevalence estimate.

**Table 2:** Characteristics of the study population according to perception category related to science communication.

Variable	Total *n* (%)	Less favorable *n* (%)	Relatively more favorable *n* (%)	*p*-value
Sex				
Female	5,713 (59.5)	3,719 (65.1)	1,994 (34.9)	<0.001
Male	3,895 (40.5)	2,693 (69.1)	1,202 (30.9)	
Age, median (IQR)	22 (20–29)	22 (20–28)	22 (20–31)	0.045
Educational level				
No formal education	42 (0.4)	35 (83.3)	7 (16.7)	0.002
Primary-secondary	1,302 (13.6)	830 (63.8)	472 (36.2)	
Technical	769 (8.0)	492 (64.0)	277 (36.0)	
Higher education	7,495 (78.0)	5,055 (67.4)	2,440 (32.6)	
Self-reported COVID-19 history				
No	9,323 (97.0)	6,229 (66.8)	3,094 (33.2)	0.358
Yes	285 (3.0)	183 (64.2)	102 (35.8)	
Country of residence				
Peru	4,739 (49.3)	3,124 (65.9)	1,615 (34.1)	<0.001
Chile	828 (8.6)	541 (65.3)	287 (34.7)	
Paraguay	685 (7.1)	425 (62.0)	260 (38.0)	
Mexico	708 (7.4)	468 (66.1)	240 (33.9)	
Colombia	493 (5.1)	362 (73.4)	131 (26.6)	
Bolivia	450 (4.7)	323 (71.8)	127 (28.2)	
Panama	443 (4.6)	287 (64.8)	156 (35.2)	
Ecuador	321 (3.3)	241 (75.1)	80 (24.9)	
Costa Rica	298 (3.1)	169 (56.7)	129 (43.3)	
El Salvador	239 (2.5)	170 (71.1)	69 (28.9)	
Honduras	199 (2.1)	144 (72.4)	55 (27.6)	
Other countries	205 (2.1)	158 (77.1)	47 (22.9)	

IQR: interquartile range. Percentages in the perception columns are row percentages. The relatively more favorable category was defined analytically as the upper tertile of the observed total score distribution.

In the crude analysis, the relatively more favorable perception category was associated with sex, age, educational level, and country of residence. In the adjusted analysis, men showed a lower prevalence of the relatively more favorable perception category than women (aPR = 0.88; 95% CI: 0.83–0.94; *p* < 0.001). Age showed a small positive association when modeled per 10-year increase (aPR = 1.04; 95% CI: 1.01–1.06; *p* = 0.002). Compared with participants with no formal education, those with primary/secondary education (aPR = 2.10; 95% CI: 1.09–4.07; *p* = 0.027) and technical education (aPR = 2.07; 95% CI: 1.07–4.02; *p* = 0.032) showed a higher prevalence of the relatively more favorable perception category, whereas the association for higher education was borderline (aPR = 1.92; 95% CI: 0.99–3.70; *p* = 0.052). Self-reported history of COVID-19 was not associated with the outcome (aPR = 1.08; 95% CI: 0.93–1.27; *p* = 0.312).

Regarding country of residence, and using Peru as the reference category, higher prevalences of the relatively more favorable perception category were observed in Paraguay (aPR = 1.13; 95% CI: 1.02–1.26; *p* = 0.021) and Costa Rica (aPR = 1.25; 95% CI: 1.09–1.43; *p* = 0.002). Lower prevalences were observed in Colombia (aPR = 0.79; 95% CI: 0.68–0.92; *p* = 0.002), Bolivia (aPR = 0.85; 95% CI: 0.73–0.99; *p* = 0.036), Ecuador (aPR = 0.76; 95% CI: 0.62–0.92; *p* = 0.006), and Other countries (aPR = 0.68; 95% CI: 0.53–0.88; *p* = 0.003) (Table 3).

**Table 3:** Factors associated with the relatively more favorable perception category related to science communication.

Variable	Crude model			Adjusted model		
	PR	95% CI	*p*-value	PR	95% CI	*p*-value
Sex						
Female	Ref			Ref		
Male	0.88	0.83–0.94	<0.001	0.88	0.83–0.94	<0.001
Age, per 10-year increase	1.04	1.02–1.07	<0.001	1.04	1.01–1.06	0.002
Educational level						
No formal education	Ref			Ref		
Primary-secondary	2.18	1.10–4.29	0.025	2.10	1.09–4.07	0.027
Technical	2.16	1.09–4.28	0.027	2.07	1.07–4.02	0.032
Higher education	1.95	0.99–3.84	0.053	1.92	0.99–3.70	0.052
Self-reported COVID-19 history						
No	Ref			Ref		
Yes	1.08	0.92–1.26	0.349	1.08	0.93–1.27	0.312
Country of residence						
Peru	Ref			Ref		
Chile	1.02	0.92–1.13	0.743	1.00	0.91–1.11	0.944
Paraguay	1.11	1.00–1.24	0.042	1.13	1.02–1.26	0.021
Mexico	0.99	0.89–1.11	0.925	1.02	0.91–1.14	0.773
Colombia	0.78	0.67–0.91	0.001	0.79	0.68–0.92	0.002
Bolivia	0.83	0.71–0.96	0.015	0.85	0.73–0.99	0.036
Panama	1.03	0.91–1.18	0.627	1.07	0.94–1.23	0.298
Ecuador	0.73	0.60–0.89	0.002	0.76	0.62–0.92	0.006
Costa Rica	1.27	1.11–1.46	0.001	1.25	1.09–1.43	0.002
El Salvador	0.85	0.69–1.04	0.109	0.87	0.71–1.06	0.172
Honduras	0.81	0.65–1.02	0.072	0.83	0.66–1.04	0.112
Other countries	0.67	0.52–0.87	0.002	0.68	0.53–0.88	0.003

PR: prevalence ratio; CI: confidence interval. Crude and adjusted PRs were estimated using generalized linear models from the Poisson family with log link and robust variance. The adjusted model included sex, age, educational level, self-reported COVID-19 history, and country of residence. Age was modeled per 10-year increase. Reference categories were female sex, no formal education, no self-reported history of COVID-19, and Peru.

In sensitivity analyses, the direction of the association for sex remained consistent when using the continuous score, the median cut-off, the 75th percentile cut-off, and the model excluding Peru. Several country-level contrasts also remained directionally consistent, whereas associations involving age and educational level varied depending on the operational definition of the outcome. These findings are presented in Supplementary Material 2.

## Discussion

This study aimed to identify factors associated with a relatively more favorable perception category related to science communication during the COVID-19 pandemic in a multicountry sample of Latin American adults. The main findings indicate that the relatively more favorable perception category was associated with sex, educational level, and country of residence, whereas age showed only a minimal association. These results are consistent with the broader literature showing that, during an infodemic, public responses to science communication depend not only on the factual accuracy of the information but also on trust in the source, message clarity, interpretability, and the broader informational environment in which messages circulate (8–10, 23, 24). From a theoretical perspective, this supports the view that science communication during public health emergencies is not simply a process of information transmission, but a socially mediated process shaped by credibility, uncertainty, prior trust, and the capacity of audiences to interpret scientific messages (1, 3, 5). Because the questionnaire was specifically developed for this study, these findings should be interpreted as reflecting an exploratory measurement approach to perceptions related to science communication rather than a fully validated latent construct.

One of the main findings was that men showed a lower prevalence of the relatively more favorable perception category than women. This result is plausible in light of previous evidence indicating that women tended to perceive COVID-19 as a more serious health threat, were more likely to support preventive measures, and generally reported higher adherence to protective behaviors during the pandemic (25). In addition, studies on digital health literacy and online health information-seeking have shown that gender may influence how people search for, appraise, and use health information, which may partly help explain the more favorable perceptions observed among women in our sample (26). Rather than reflecting an intrinsic difference, this pattern may be interpreted as the expression of gendered differences in risk perception, attentiveness to health information, and engagement with public health messaging during a period of high uncertainty.

Educational level also showed a consistent association with the outcome. Participants with formal education had a higher prevalence of the relatively more favorable perception category than those with no formal education. This finding is conceptually coherent with the literature on health literacy, which suggests that individuals with greater educational resources are better positioned to identify reliable information, interpret complex or changing recommendations, and navigate informational environments characterized by competing narratives and misinformation (27, 28). In the context of COVID-19, these capacities may have been especially relevant because scientific information was rapidly evolving and often communicated through multiple channels of variable quality. Our findings therefore support the idea that favorable perceptions of science communication are partly shaped by the cognitive and informational resources needed to process evidence under conditions of uncertainty. At the same time, the magnitude of the observed associations was modest, suggesting that education is only one component of a broader set of social and contextual determinants.

The between-country differences observed in this study should be interpreted cautiously, but they are unlikely to be random. Compared with Peru, higher prevalences of the relatively more favorable perception category were observed in Paraguay and Costa Rica, whereas lower prevalences were observed in Colombia, Bolivia, Ecuador, and the grouped category of Other countries. These findings should not be interpreted as fixed country rankings or intrinsic national traits. Rather, they probably reflect differences in information ecosystems, institutional trust, public communication strategies, and the structural characteristics of health systems across the region. Latin America entered the pandemic with profound social inequities, segmented and fragmented health systems, and heterogeneous institutional capacities to organize coherent public health responses (20). In that setting, misinformation and distrust became major barriers to effective communication, including in relation to vaccination and other public health measures (29). In addition, false information circulated widely across Latin American digital environments during the pandemic, contributing to confusion and uncertainty in public debate (30). WHO-led work on infodemic management also emphasizes that local communication environments, digital access, media ecosystems, and institutional coordination shape how populations encounter and interpret health information (5, 15). These processes unfolded in a region that was already experiencing a severe and uneven pandemic burden, under conditions of accumulated vulnerability and institutional strain (22, 31, 32). From this perspective, the country-level differences observed here are better understood as markers of contextual heterogeneity than as isolated national effects.

Although age was statistically associated with the outcome, the magnitude of this association was minimal. Substantively, this suggests that age was not a major determinant of the relatively more favorable perception category in this sample. This result should be interpreted in light of the study design, since the survey was administered online and the sample was predominantly young, with a median age of 22 years. Under these conditions, age-related differences may have been attenuated by the selective participation of individuals who were already digitally connected and able to engage with an online questionnaire. Similarly, the absence of a relevant association with self-reported history of COVID-19 suggests that perceptions of science communication may have depended more on the surrounding informational context than on prior direct experience with the disease itself.

From a public health perspective, these findings reinforce that science communication is not received uniformly across populations. During health emergencies, effective communication depends on whether messages are perceived as understandable, credible, consistent, and socially relevant, and on whether they are delivered through channels that the population considers trustworthy (8–10, 23, 24, 33). In practical terms, our results suggest that communication strategies in Latin America should pay particular attention to educational inequalities and to the uneven informational conditions in which scientific messages are interpreted. This implies the need for plain language, transparent communication of uncertainty, consistency across spokespersons, and more active responses to misinformation circulating through regional digital networks. It also implies moving from reactive correction of false claims toward broader infodemic management strategies, including monitoring circulating narratives, tailoring messages to local contexts and literacy levels, and strengthening partnerships between public health authorities, scientific actors, media, and digital platforms (5, 7).

This study has several strengths. It addresses a relevant but still insufficiently explored outcome in the Latin American context, includes a large multicountry sample, and examines perceptions related to science communication during a critical stage of the pandemic. However, the findings should be interpreted in light of important limitations. First, the non-probabilistic snowball sampling strategy limits representativeness and likely favored the inclusion of younger, more educated, and more digitally connected participants. Second, Peru contributed nearly half of the sample, which may have influenced country comparisons. Although the sensitivity analysis excluding Peru showed that several associations remained directionally consistent, the imbalance in country contributions still limits the interpretation of pooled and country-specific estimates. Third, because the relatively more favorable perception category was operationalized using the upper tertile of the total score distribution, the proportion classified in that category should not be interpreted as a population prevalence estimate, but rather as an analytical classification used for association analyses. Moreover, because the study had a large sample size, statistically significant associations with small magnitudes should be interpreted cautiously in terms of practical or public health relevance. Fourth, the cross-sectional design precludes causal inference. Finally, although the questionnaire showed acceptable internal consistency, internal consistency alone does not establish full measurement validity, and additional evidence on dimensionality and other measurement properties would be needed for a more robust psychometric evaluation (34). In addition, no formal assessment of structural validity, item-level performance beyond alpha-if-item-deleted, test–retest reliability, or cross-country measurement equivalence was performed. Therefore, country-level comparisons should be interpreted as exploratory associations with the observed composite score rather than as definitive differences in a validated latent construct across countries. Some items may also reflect related but not identical domains, such as perceived credibility, clarity of communication, perceived usefulness of spokespersons, and exposure to questionable content. Therefore, the questionnaire should be considered exploratory, and future studies should examine its content validity more formally, structural validity, reproducibility, and measurement performance across different populations and country contexts.

## Conclusion

The findings suggest that the relatively more favorable perception category related to science communication during the COVID-19 pandemic was socially patterned in this Latin American sample, particularly according to sex, educational level, and country of residence. These results support the view that science communication in public health is not passively received, but is filtered through unequal social, educational, and contextual conditions. Given the exploratory nature of the questionnaire and the analytical definition of the outcome, these findings should be interpreted as hypothesis-generating rather than as population estimates of favorable perceptions. Future research should build on these findings using more representative samples and stronger psychometric evaluation of measurement tools, while public health practice should move toward more equitable, context-sensitive, and trust-oriented communication strategies.

## Data Availability

The raw data supporting the conclusions of this article will be made available by the authors, without undue reservation.
