# Supplementary material for: Factors associated with perceptions of science communication in Latin America during the COVID-19 pandemic
Source: Front Public Health. 2026 Aug 20;14:1853486. doi: 10.3389/fpubh.2026.1853486 (PMC13539601; doi:10.3389/fpubh.2026.1853486)
Supplement: Supplementary file 1 [file Data_Sheet_1.docx]

**Supplementary Material 1. Questionnaire items, response options, scoring scheme, and conceptual mapping**

**Response options and scoring**

Each item was answered using a five-point Likert-type response scale: strongly disagree, disagree, neither agree nor disagree, agree, and strongly agree. Responses were coded from 1 to 5, respectively. The total exploratory composite score was calculated as the sum of the 11 items, with possible values ranging from 11 to 55.

No items were reverse-coded in the main scoring scheme. Although some items referred to exposure to false or dubious information, pessimistic media coverage, or perceived harm from media information, the original scoring scheme was retained because all items showed positive item-test correlations and positive loadings on the first factor in the exploratory factor analysis. Therefore, the total score should be interpreted as an exploratory composite measure of perceptions and experiences related to science communication, rather than as a fully validated unidimensional scale.

**Analytical categorization**

For the main analysis, the upper tertile of the observed total score distribution was used to classify participants into the relatively more favorable perception category. Participants in the lower and middle tertiles were grouped into the less favorable category. This cut-off was used only as an analytical threshold and should not be interpreted as a validated psychometric, clinical, or population-based definition of favorable perception.

| **Supplementary Table S1. Questionnaire items, scoring scheme, and conceptual mapping** | | | |
| --- | --- | --- | --- |
| **Item** | **Questionnaire item** | **Scoring** | **Main conceptual domain** |
| 1 | Do you think that the information being disseminated about supposed remedies or cures for the coronavirus is based on verified scientific sources? | 1–5 | Perceived credibility of information on remedies or cures |
| 2 | Do you think that the information being disseminated about how the coronavirus originated is based on verified scientific sources? | 1–5 | Perceived credibility of information on virus origin |
| 3 | Have you come across false or dubious scientific information about the coronavirus? | 1–5 | Exposure to false or dubious scientific information |
| 4 | Do you believe that the news media provide adequate scientific information about COVID-19? | 1–5 | Perceived adequacy of scientific information in news media |
| 5 | Do you think the language used by online media to inform the public is understandable? | 1–5 | Clarity and understandability of media language |
| 6 | Based on what the media shows you daily, do you perceive that most news is bad or pessimistic? | 1–5 | Perceived negative or pessimistic tone of media coverage |
| 7 | Would you like to have a source that confirms or refutes the news you hear or see in the media? | 1–5 | Perceived need for fact-checking or verification sources |
| 8 | Do you believe the doctors interviewed helped you understand/clarify the scientific news about COVID-19? | 1–5 | Perceived usefulness of physicians as scientific communicators |
| 9 | Do you believe that the other scientists interviewed helped you understand/clarify the scientific news about COVID-19? | 1–5 | Perceived usefulness of scientists as scientific communicators |
| 10 | Has the scientific information provided by the government been useful in combating the coronavirus? | 1–5 | Perceived usefulness of government-provided scientific information |
| 11 | Did the information provided by the media affect you or cause you any problems or harm at any point during the pandemic? | 1–5 | Perceived personal impact or harm from media information |
| All items were scored from 1 to 5 according to the original coding scheme defined by the research team. No items were reverse-coded in the main scoring scheme. The conceptual domains shown here should not be interpreted as formally validated subscales. | | | |
