# Supplementary material for: Factors associated with perceptions of science communication in Latin America during the COVID-19 pandemic
Source: Front Public Health. 2026 Aug 20;14:1853486. doi: 10.3389/fpubh.2026.1853486 (PMC13539601; doi:10.3389/fpubh.2026.1853486)
Supplement: Supplementary file 2 [file Data_Sheet_2.docx]

**Supplementary Material 2. Exploratory factor analysis, sensitivity analyses, and supplementary figure**

This supplementary material presents the exploratory factor analysis of the 11-item questionnaire and sensitivity analyses conducted to assess the robustness of the main findings.

| **Supplementary Table S1. Exploratory factor analysis of the 11-item questionnaire** | | |
| --- | --- | --- |
| **Item** | **Factor 1 loading** | **Uniqueness** |
| Item 1 | 0.320 | 0.898 |
| Item 2 | 0.485 | 0.765 |
| Item 3 | 0.644 | 0.586 |
| Item 4 | 0.545 | 0.703 |
| Item 5 | 0.660 | 0.565 |
| Item 6 | 0.578 | 0.666 |
| Item 7 | 0.734 | 0.461 |
| Item 8 | 0.813 | 0.339 |
| Item 9 | 0.824 | 0.321 |
| Item 10 | 0.665 | 0.557 |
| Item 11 | 0.412 | 0.830 |
| Principal-component factor analysis. The first factor had an eigenvalue of 4.31 and explained 39.2% of the variance. Three factors had eigenvalues greater than 1 and together explained 62.1% of the variance. | | |

| **Supplementary Table S2. Sensitivity analysis using the total score as a continuous outcome** | | | |
| --- | --- | --- | --- |
| **Variable** | **β coefficient** | **95% CI** | **p-value** |
| Male | -0.73 | -1.05 to -0.40 | <0.001 |
| Age, per 10-year increase | -0.12 | -0.27 to 0.02 | 0.096 |
| Primary-secondary | 4.54 | 1.78 to 7.30 | 0.001 |
| Technical | 4.41 | 1.62 to 7.20 | 0.002 |
| Higher education | 4.47 | 1.74 to 7.20 | 0.001 |
| Self-reported COVID-19 history, yes | -0.75 | -1.86 to 0.36 | 0.183 |
| Chile | 1.01 | 0.45 to 1.57 | <0.001 |
| Paraguay | 0.79 | 0.12 to 1.46 | 0.021 |
| Mexico | 0.48 | -0.12 to 1.08 | 0.119 |
| Colombia | 0.55 | -0.08 to 1.17 | 0.088 |
| Bolivia | -0.14 | -0.88 to 0.59 | 0.699 |
| Panama | 1.44 | 0.78 to 2.09 | <0.001 |
| Ecuador | -0.15 | -0.95 to 0.65 | 0.706 |
| Costa Rica | 1.65 | 0.77 to 2.53 | <0.001 |
| El Salvador | -0.88 | -1.99 to 0.22 | 0.117 |
| Honduras | -0.43 | -1.63 to 0.77 | 0.479 |
| Other countries | -1.50 | -2.68 to -0.32 | 0.013 |
| Linear regression with robust standard errors. Reference categories were female sex, no formal education, no self-reported history of COVID-19, and Peru. | | | |

The sensitivity analysis excluding Peru showed that the direction of the association for sex remained consistent. Several country-level contrasts also remained directionally consistent. However, estimates for educational level were unstable because of sparse data in the no formal education reference category after excluding Peru; therefore, these estimates were not interpreted substantively.

| **Supplementary Table S3. Sensitivity analysis using the median cut-off** | | | |
| --- | --- | --- | --- |
| **Variable** | **Adjusted PR** | **95% CI** | **p-value** |
| Male | 0.91 | 0.88–0.95 | <0.001 |
| Age, per 10-year increase | 1.00 | 0.99–1.02 | 0.699 |
| Primary-secondary | 2.49 | 1.40–4.43 | 0.002 |
| Technical | 2.43 | 1.36–4.33 | 0.003 |
| Higher education | 2.44 | 1.38–4.33 | 0.002 |
| Self-reported COVID-19 history, yes | 1.00 | 0.90–1.12 | 0.976 |
| Chile | 1.06 | 0.99–1.13 | 0.084 |
| Paraguay | 1.14 | 1.07–1.22 | <0.001 |
| Mexico | 1.01 | 0.94–1.09 | 0.842 |
| Colombia | 0.91 | 0.83–1.00 | 0.062 |
| Bolivia | 0.99 | 0.90–1.08 | 0.768 |
| Panama | 1.13 | 1.04–1.23 | 0.004 |
| Ecuador | 0.80 | 0.70–0.91 | 0.001 |
| Costa Rica | 1.15 | 1.05–1.27 | 0.003 |
| El Salvador | 0.88 | 0.77–1.01 | 0.073 |
| Honduras | 0.92 | 0.80–1.07 | 0.281 |
| Other countries | 0.87 | 0.75–1.01 | 0.077 |
| Poisson regression with log link and robust variance. The outcome was defined using the median of the observed total score distribution. | | | |

| **Supplementary Table S4. Sensitivity analysis using the 75th percentile cut-off** | | | |
| --- | --- | --- | --- |
| **Variable** | **Adjusted PR** | **95% CI** | **p-value** |
| Male | 0.84 | 0.79–0.90 | <0.001 |
| Age, per 10-year increase | 1.05 | 1.02–1.08 | <0.001 |
| Primary-secondary | 1.74 | 0.90–3.35 | 0.099 |
| Technical | 1.79 | 0.93–3.47 | 0.083 |
| Higher education | 1.59 | 0.83–3.05 | 0.165 |
| Self-reported COVID-19 history, yes | 1.10 | 0.92–1.32 | 0.274 |
| Chile | 0.99 | 0.88–1.11 | 0.887 |
| Paraguay | 1.16 | 1.03–1.31 | 0.013 |
| Mexico | 0.99 | 0.87–1.13 | 0.884 |
| Colombia | 0.80 | 0.68–0.95 | 0.012 |
| Bolivia | 0.73 | 0.61–0.89 | 0.001 |
| Panama | 0.96 | 0.81–1.13 | 0.633 |
| Ecuador | 0.71 | 0.57–0.90 | 0.004 |
| Costa Rica | 1.22 | 1.04–1.44 | 0.015 |
| El Salvador | 0.89 | 0.71–1.12 | 0.338 |
| Honduras | 0.80 | 0.61–1.05 | 0.102 |
| Other countries | 0.69 | 0.52–0.92 | 0.011 |
| Poisson regression with log link and robust variance. The outcome was defined using the 75th percentile of the observed total score distribution. | | | |
